# Facilitating students' project work with online collaborative support: A case from Business Administration Discipline at Hong Kong Institute of Vocational Education (Lee Wai Lee)

**DOI:** 10.1186/2193-1801-4-S2-P4

**Published:** 2015-11-27

**Authors:** Janet Man-wai Cheung, Emma Yim-ha Leung, Candy Mei-ting Leung

**Affiliations:** Research Support Unit, Vocational Training Council, Hong Kong; Department of Business Administration, Hong Kong Institute of Vocational Education (Lee Wai Lee), Hong Kong; Web-based VEP Development Project Office, Languages Planning and Development Unit, Vocational Training Council, Hong Kong

## Background

'Generation Z' [1] students are immersed in digital technology, which has 'a profound impact on their personalities, including their attitudes and approach to learning' [2]. This 'forces a change in the model of pedagogy... based on collaboration' [3].

Collaborative learning is a social act through which learning occurs [4]. Over 90% of students in a survey found group work with peers was the most engaging [5].

To facilitate collaborative learning, online drives and communication tools are useful for matters such as time-saving, flexible access, and ease of sharing [6].

Moodle, the official learning management platform of the VTC, facilitates sharing of learning materials. Its discussion forum can support asynchronous exchange of ideas.

The aim of the study is to explore whether online collaborative support is an effective means of facilitating students' project work.

## Methods

Four classes studying Business Administration Discipline at the Hong Kong Institute of Vocational Education (Lee Wai Lee) took a module on 'Crisis Management' in semester 2 of the academic year 2014-2015. They needed to share preliminary research findings in a Moodle forum, and save project reports and presentation slides to an online drive. In addition to tracking anonymous data from the online platforms, a questionnaire survey was given to 91 students and an interview was conducted with 22 students. The teacher was also interviewed. The aim of the surveys was to collect respondents' reflections on their online participation, reasons for participation, and the usefulness of the technology.

## Results

The results of this pilot study are summarised in two parts: students' online engagement and feedback from the perception survey.

*Part 1: Students' Online Engagement*

As reflected by the learning analytics, 352.78 page views per day were recorded during the semester, equivalent to 10 page views per visit. On average, students spent 4 minutes 17 seconds on each visit.

The three days when the highest numbers of users were recorded were in the last weeks of the semester. Another peak was recorded at the beginning of the semester, when the online platforms were introduced to the classes.

The top three most popular Moodle activities recorded 1394 views, covering a quarter of the total. 152 posts were shared in the discussion forums, in which different perspectives on the case studies were raised.

*Part 2: Feedback from the Perception Survey*

A perception survey in the form of a questionnaire was disseminated immediately after the students' presentations. The survey results indicate that 'course requirement' and 'interest to learn' were students' main motivations to complete the online activities (roughly 40%). The convenience of the technology was an attraction for 23.6% of the respondents.

The most frequent online work was discussion via WhatsApp (over 60%), followed by other project related work such as web searches and Moodle discussion (38%).

More than half the respondents reflected that WhatsApp and the readings in Moodle were useful for their study. The online drive was the third most useful feature (44.4%).

The interviewees were positive about using technology in their studies: WhatsApp was used for daily communication such as project reminders; the Moodle forum was used for cross-class and cross-team exchange; the online drive was the working platform for project documents. They found this learning mode time-saving, and some aspects, such as different views collected from forums, were helpful for comprehension. However, they found Moodle was not user-friendly when logging in or sending large files.

The teacher shared strategies in the interview: planning online activities well and demonstrating the use of technologies at the beginning of the semester, and giving feedback in the online platforms during the project period. Overall, this teaching method was found to be effective for project-based modules.

## Conclusions

Teachers' demonstrations and setting online work as a module requirement are important in engaging students online. The inclusion of communication tools such as WhatsApp and Moodle was essential and well-liked for collaborative project work. When ranking the usefulness of the technologies for study, WhatsApp, readings in Moodle and the online drive were the three most popular choices.

In summary, incorporating an online drive and online communication tools into daily learning is useful for project-based tasks.
